# Drugs for some but not all: inequity within community health worker teams during introduction of integrated community case management

**DOI:** 10.1186/1472-6963-14-S1-S1

**Published:** 2014-05-12

**Authors:** Hannah Faye G Mercader, Teddy Kyomuhangi, Denise L Buchner, Jerome Kabakyenga, Jennifer L Brenner

**Affiliations:** 1 Faculty of Medicine, University of Calgary, Calgary, Alberta, T2N 1N4, Canada; 2 Faculty of Medicine, Mbarara University of Science and Technology, Mbarara, Uganda

**Keywords:** community health worker, equity, health promotion, integrated community case management, motivation, Uganda, travailleurs en santé communautaire, équité, promotion de la santé, gestion des cas intégrée en milieu communautaire, motivation, Ouganda

## Abstract

**Background:**

The Ugandan health system now supports integrated community case management (iCCM) by community health workers (CHWs) to treat young children ill with fever, presumed pneumonia, and diarrhea. During an iCCM pilot intervention study in southwest Uganda, two CHWs were selected from existing village teams of two to seven CHWs, to be trained in iCCM. Therefore, some villages had both ‘basic CHWs’ who were trained in standard health promotion and ‘iCCM CHWs’ who were trained in the iCCM intervention. A qualitative study was conducted to investigate how providing training, materials, and support for iCCM to some CHWs and not others in a CHW team impacts team functioning and CHW motivation.

**Methods:**

In 2012, iCCM was implemented in Kyabugimbi sub-county of Bushenyi District in Uganda. Following seven months of iCCM intervention, focus group discussions and key informant interviews were conducted alongside other end line tools as part of a post-iCCM intervention study. Study participants were community leaders, caregivers of young children, and the CHWs themselves (‘basic’ and ‘iCCM’). Qualitative content analysis was used to identify prominent themes from the transcribed data.

**Results:**

The five main themes observed were: motivation and self-esteem; selection, training, and tools; community perceptions and rumours; social status and equity; and cooperation and team dynamics. ‘Basic CHWs’ reported feeling hurt and overshadowed by ‘iCCM CHWs’ and reported reduced self-esteem and motivation. iCCM training and tools were perceived to be a significant advantage, which fueled feelings of segregation. CHW cooperation and team dynamics varied from area to area, although there was an overall discord amongst CHWs regarding inequity in iCCM participation. Despite this discord, reasonable personal and working relationships within teams were retained.

**Conclusions:**

Training and supporting only some CHWs within village teams unexpectedly and negatively impacted CHW motivation for ‘basic CHWs’, but not necessarily team functioning. A potential consequence might be reduced CHW productivity and increased attrition. CHW programmers should consider minimizing segregation when introducing new program opportunities through providing equal opportunities to participate and receive incentives, while seeking means to improve communication, CHW solidarity, and motivation.

## Abstract (fr)

**Résumé:**

**Contexte**

Le système de santé ougandais soutient aujourd’hui la gestion des cas intégrée en milieu communautaire – par des travailleurs en santé communautaire – pour le traitement de la fièvre, de la pneumonie présumée et de la diarrhée chez les enfants. Dans le cadre de l’étude de l’intervention pilote de gestion des cas intégrée dans le sud de l’Ouganda, deux membres des équipes de travailleurs en santé communautaire de chaque village (qui comptent de deux à sept travailleurs chacune) ont été choisis pour recevoir de la formation en gestion des cas intégrée. Certains villages comptaient donc à la fois des travailleurs « types », formés en promotion de la santé, et des travailleurs « spéciaux », formés aux interventions en gestion des cas intégrée. Une étude qualitative a été réalisée pour déterminer la manière dont l’apport de formation, de matériel et de soutien en matière de gestion des cas intégrée à une partie seulement des travailleurs en santé communautaire se répercute sur le fonctionnement et la motivation des équipes.

**Méthodes**

En 2012, la gestion des cas intégrée en milieu communautaire a été déployée dans le sous-comté de Kyabugimbi (district de Bushenyi), en Ouganda. Au terme d’une intervention de sept mois en gestion des cas intégrée, on a mis sur pied des groupes de discussion, réalisé des entrevues avec des intervenants clés et mis en place d’autres outils d’évaluation finale dans le cadre d’une étude post-intervention. Les participants à l’étude étaient des dirigeants communautaires, des fournisseurs de soins destinés aux jeunes enfants ainsi que des travailleurs « types » et des travailleurs « spéciaux ». On a aussi procédé à une analyse qualitative du contenu pour déterminer les principaux thèmes étant ressortis des données transcrites.

**Résultats**

Les cinq principaux thèmes qui sont ressortis de l’analyse sont : la motivation et l’estime de soi; la sélection, la formation et les outils; les perceptions et les rumeurs circulant dans les collectivités; le statut social et l’équité; la coopération et la dynamique au sein des équipes. Les travailleurs « types» ont avoué s’être sentis blessés et éclipsés par les travailleurs « spéciaux ». Ils ont reconnu être moins motivés et avoir une moins bonne estime d’eux-mêmes. La formation et les outils en matière de gestion des cas intégrée constituaient pour la plupart des travailleurs un important avantage, ce qui a alimenté le sentiment de ségrégation des travailleurs « types ». Si la collaboration et la dynamique au sein des équipes de travailleurs varient d’un endroit à l’autre, la désapprobation à l’égard des disparités de participation à la gestion des cas intégrée, elle, est généralisée. Pourtant, des rapports interpersonnels et professionnels acceptables ont été maintenus au sein de toutes les équipes.

Si l’apport d’une formation et d’un soutien à certains travailleurs uniquement a eu des répercussions négatives imprévues sur la motivation des travailleurs « types », il n’a pas nécessairement altéré le fonctionnement des équipes. Une conséquence possible serait une baisse de la productivité des travailleurs et une augmentation de l’attrition. Les gestionnaires responsables des travailleurs en santé communautaire doivent réduire la ségrégation au minimum lorsqu’ils lancent de nouveaux programmes et veiller à offrir les mêmes occasions de participation et les mêmes mesures incitatives à tous les travailleurs, tout en cherchant des façons d’améliorer la communication, la solidarité et la motivation au sein des équipes.

## Background

An estimated 7.2 million children under the age of five years die each year and nearly half of these deaths occur in sub-Saharan Africa [1]. Most under-five deaths occur from preventable illnesses such as pneumonia, diarrhea, and malaria [2]. Child deaths often occur where there are shortages of health workers, particularly in rural and resource-poor settings. To address this shortage of health professionals, many countries have implemented a task shifting model of health service delivery, in which health care tasks are delegated to less specialized health workers [3]. In 2010, the Uganda Ministry of Health (MoH) formalized a policy to use a lay community health worker (CHW) program for health promotion and additionally, for some of these CHWs to provide integrated community case management (iCCM) [4]. ICCM involves training and equipping CHWs to assess and provide simple treatment for sick children under five years old: Coartem (artemether/lumefantrine) for fever, amoxicillin for presumed pneumonia, and zinc and oral rehydration salts for diarrhea.

Research suggests that iCCM can be effective in rural sub-Saharan Africa [5-7]. However, the operational logistics of how to best implement and scale-up iCCM programs remain poorly studied, with many gaps in our understanding of the optimal approaches [8,9]. Retention of a critical mass of CHWs is particularly important to keep programs sustainable and cost-effective. Since CHWs often work as volunteers, their motivation and willingness must be nurtured in order for such programs to continue and succeed. Factors that may impede CHW motivation are critical to understand and address before iCCM programs are rolled out nationally [10-12].

Healthy Child Uganda (HCU) is a Ugandan-Canadian university partnership that works to improve maternal, newborn, and child health outcomes in Southwest Uganda through a number of different health programs, with emphasis on CHW support. From 2010 to 2012, the HCU team partnered with the Uganda MoH to conduct a pilot study that assessed whether iCCM provided by trained lay volunteer CHWs could increase access to care for sick young children. Using the government iCCM guidelines, the HCU team was responsible for implementing CHW training in iCCM as well as conducting the field research. The intervention showed a significantly increased proportion of children under the age of five with fever, presumed pneumonia, and diarrhea who received treatment, compared with a control group [13]. However, anecdotal reports from CHW supervisors suggested that issues of segregation and demotivation within some CHW teams might have emerged during project implementation. It is important to investigate such issues to better understand optimal approaches for creating community intervention programs, particularly in iCCM. A scan of the literature revealed no recent studies examining similar themes. Therefore, a qualitative study was designed to more objectively understand if and how providing training, materials, and support for iCCM to some CHWs and not others in a CHW team, impacts team functioning and CHW motivation.

## Methods

### Study setting

Kyabugimbi sub-county of Bushenyi District, which consists of 98 villages, was selected for this pilot study because of its rural location and limited infrastructure, and because the HCU team had strong established relationships in the district. In 2010, 288 CHWs were initially selected by their respective villages and received five days of ‘basic CHW’ training as per Ministry of Health CHW guidelines [14]. One ‘basic CHW’ was selected for every 25-30 households, therefore each village selected between two and seven ‘basic CHWs’ depending on the village size. The five-day ‘basic CHW’ training course included introduction to the CHW role, village health reporting, and health promotion related to water/sanitation, nutrition, vaccination, and common illness prevention. Upon completion of training, ‘basic CHWs’ received a training certificate, a badge, a T-shirt, a canvas bag, a job-aid, registers, and health promotion materials.

**Figure 1:**
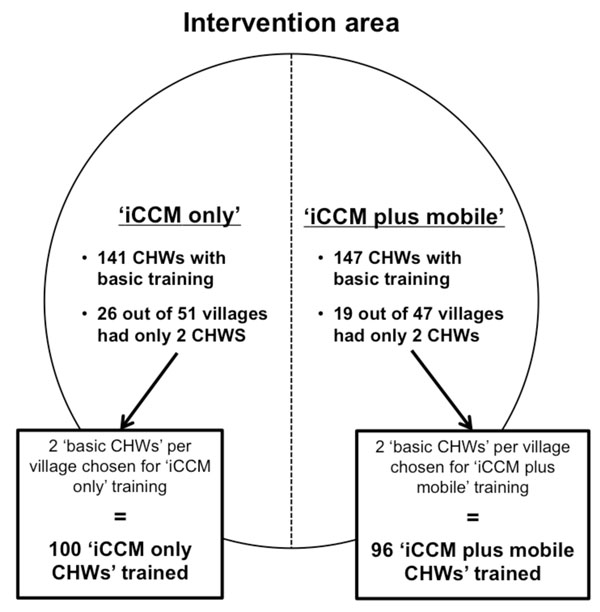
**CHW selection method for ‘iCCM only’ or ‘iCCM plus mobile’ training** In the iCCM study intervention area, each village had a total of two to seven CHWs working in a team. Two CHWs from each team per village were then chosen to be trained in and provide iCCM, resulting in 68% of all CHWs to be trained. About half of the villages in the intervention area were also designated as ‘iCCM plus mobile’, where their ‘iCCM CHWs’ were trained in both iCCM and mobile phones. In the intervention area, 45 of 98 villages (46%) had all CHWs in their villages trained in iCCM, whereas the remaining villages had one or more remaining ‘basic CHWs’ who conducted standard CHW tasks.

In March 2012, the ‘basic CHWs’ selected 196 individuals from amongst themselves to also be trained as ‘iCCM CHWs’. Two CHWs were chosen from each village, regardless of size, as per government recommendations [4]. Although no specific criteria were given for selecting ‘iCCM CHWs’, geographical location of CHWs within villages was often a main factor for selection. About half of villages had only two ‘basic CHWs’ to begin with; therefore these villages could select all their CHWs to be trained as ‘iCCM CHWs’. However villages with more than two ‘basic CHWs’ had one or more CHWs that remained as a ‘basic CHW’. Each new ‘iCCM CHW’ attended an additional five-day iCCM training course, which emphasized assessment and treatment of children with fever, presumed pneumonia, and diarrhea using an algorithm, and record-keeping of patient encounters. Each ‘iCCM CHW’ received a training certificate, a canvas bag, a respiratory timer, a sick child job-aid, a wooden medicine box, registers, referral forms, and a starter supply of medicines. About half of the villages in Kyabugimbi were randomly selected to also be part of a second intervention study arm called the ‘iCCM plus mobile’ group, which involved using mobile phones to support iCCM. The ‘iCCM CHWs’ in these villages received five more days of training (i.e. total of 10 additional days) covering mobile phone use, and were provided with one mobile phone, a solar charger, and a lamp, in addition to the usual iCCM package. Figure 1 summarizes the selection method for training ‘iCCM CHWs’.

Overall, the pilot study had three categories of CHWs: 1) 92 ‘basic CHWs’ who provided basic health promotion, 2) 100 ‘iCCM only CHWs’ who provided health promotion and iCCM, and 3) 96 ‘iCCM plus mobile CHWs’ who provided health promotion and iCCM with mobile phone technology. Table 1 compares major tasks for the different CHW roles. All CHWs trained in iCCM, whether provided with or without a mobile phone, are collectively referred to as ‘iCCM CHWs’ in this paper. Regardless of category, all CHWs were assigned specific tasks and expected to attend monthly meetings. All CHWs were volunteers and did not receive salaries or financial incentives other than a US$1 equivalent transport stipend for each day of training.

**Table 1:** Comparison of major tasks for different CHW roles

‘Basic CHW’	‘iCCM only CHW’	‘iCCM plus mobile CHW’
• Visit homes • Mobilize for health service utilization • Promote health through education (e.g. hygiene and sanitation) • Advise families on home management of common health conditions (e.g. ORS for diarrhea) • Follow up visits to pregnant and post-natal women and newborns • Follow up discharged and chronic patients • Distribute health commodities (e.g. mosquito nets) • Report on health indicators in villages and outbreaks	• ‘Basic CHW’ tasks • Assess sick children • Identify main symptoms and danger signs for malaria, pneumonia, and diarrhea • Support, treat, and/or refer sick children • Record patient encounters in registers • Maintain supply of essential drugs	• ‘Basic CHW’ tasks • ‘iCCM only CHW’ tasks • Record patient encounters through mobile phone application

The iCCM portion of the intervention lasted seven months during which time ‘iCCM CHWs’ provided iCCM care to sick children in their communities. Tools for this qualitative study were developed alongside other qualitative and quantitative end line data collection tools as part of a post-iCCM intervention study conducted in November 2012 [13].

### Data collection and management

Study participants were purposely selected to represent several groups: 1) Local Council Leaders (elected officials of villages), 2) caregivers (pregnant women and/or mothers with children under two years old), and 3) CHWs (‘basic’ and ‘iCCM’). In the same way that ‘iCCM CHWs’ were further categorized into those trained in ‘iCCM only’ and those trained in ‘iCCM plus mobile’, ‘basic CHWs’ were further categorized into those from ‘iCCM only’ villages and those from ‘iCCM plus mobile’ villages. These distinctions were made in order to capture the level of influence mobile phones may have had on CHW experiences. Table 2 summarizes participant characteristics.

**Table 2:** Participant characteristics

Participants	Intervention area	Data collection type	***n***	Gender
Local Council Leader	iCCM plus mobile	KII x 2	2	2 M

Caregivers	iCCM plus mobile	FGD	8	8 F

‘iCCM plus mobile CHWs’	iCCM plus mobile	FGD	8	1 M 7 F

‘iCCM only CHWs’	iCCM only	FGD x 3	24	5 M 19 F

‘Basic CHWs’	iCCM plus mobile	FGD	7	5 M 2 F

‘Basic CHWs’	iCCM only	FGD x 3	17	3 M 14 F

M = Male. F = Female.

Focus group discussion (FGD) and key informant interview (KII) tools were developed *de novo* and contained questions related to the perceptions of the different categories of CHWs. The nature of some questions varied depending on the participant group in order to allow for a variety of themes to emerge from the discussions. For example, discussions with Local Council Leaders and caregivers included more questions exploring themes of appreciation, preference, and the quality of CHW services. Discussions with CHWs involved more questions exploring themes of workload, satisfaction, motivation, and CHW team dynamics. The tools were translated from English into the local dialect (Runyankore) and subsequently back-translated to ensure accuracy.

In December 2012, three FGDs and two KIIs were conducted with the participants. Discussions were held in familiar community locations and were facilitated in the local dialect by an experienced moderator and note-taker. All sessions were audio recorded with two digital voice recorders and audio recordings were transcribed directly into English for analysis. Six additional FGDs were conducted in March/April 2013 after analysis of the first round of data collection, as saturation was not yet achieved. The first author alone used qualitative content analysis to identify prominent themes from the transcriptions. Using NVivo 9, data were organized into codes and expanded into sub-categories, if needed. Transcripts, codes, and sub-categories were then constantly compared for patterns and reorganized as suggested by Creswell [15], finally generating the five major themes described in the results below.

### Ethical considerations

Ethics approval in this study was obtained from the Mbarara University of Science and Technology Institutional Ethical Review Committee and the University of Calgary Conjoint Health Research Ethics Board. The University of British Columbia Ethical Review Committee approved the mobile phone study component. Informed written consent (through signature or thumbprint) was obtained prior to participation in all FGDs and KIIs.

## Results

The five main themes related to the study question are presented below.

### Motivation and self-esteem

Generally, CHWs in all categories were proud of their role as volunteer health workers and attributed this pride, in part, to the process of ‘basic CHW’ selection through a community vote. CHWs reported feeling trusted by community members, and these feelings of trust motivated their volunteer work.

However, ‘iCCM CHWs’ described themselves as more proactive in their CHW tasks than did ‘basic CHW’ counterparts, sometimes even foregoing personal activities in order to administer drugs to children in their communities. This increase in commitment was noted with appreciation by caregivers. ‘iCCM CHWs’ reported feeling more important and respected for their work by the community, with a stronger sense of life direction than when they were ‘basic CHWs’. This contrasted with ‘basic CHWs’ who described themselves as feeling hurt, disregarded, and “voiceless” compared to ‘iCCM CHWs’; such feelings were strongest amongst those ‘basic CHWs’ from ‘iCCM plus mobile’ villages. Though ‘basic CHWs’ expressed a desire to feel satisfied in their work, their lack of iCCM participation reportedly made them feel useless with their roles. One ‘basic CHW’ described his/her humiliation as:

*“I felt hurt as if they used me like banana leaves*, *you know when [you no longer need them] after being used [to prepare your food*, *you] throw them away. It made me hate myself.”*

### Selection, training, and tools

‘iCCM CHWs’ reported that their additional training made them feel skilled and accomplished, despite it resulting in an increase in demanding work. They reported feeling like they were making a more valuable contribution to improving community health, and even expressed an eagerness to receive more training or materials that would make them comparable to health centre workers (e.g. diagnostic tests for malaria). Meanwhile, ‘basic CHWs’ reported feeling less efficient, overshadowed, and marginalized in comparison to ‘iCCM CHWs’, describing shame in turning sick children away because of their inability to administer drugs. In particular, ‘basic CHWs’ in ‘iCCM plus mobile’ areas spoke with envy for the mobile phones given to colleagues, since they could be used for personal needs and thus substantially raise the status of ‘iCCM plus mobile CHWs’. Furthermore, ‘basic CHWs’ reported feeling disadvantaged by not receiving the iCCM training certificate, since they perceived it as a symbol of status, knowledge, and the opportunity to advance in life. As one ‘basic CHW’ stated:

*“And you see this certificate is just a paper; when the rain rains on it*, *it decays. But if you have it*, *there is a way it makes you strong and proud.”*

Miscommunication regarding training also appeared to exacerbate self-esteem issues. For example, some ‘basic CHWs’ were allegedly promised early in the intervention roll out that all CHWs would receive iCCM training; others were promised mobile phones. Some ‘basic CHWs’ also reported feeling it was unfair how ‘iCCM CHWs’ were selected within their own CHW teams, since communities had originally voted for all CHWs with the intent that they would be equals. Despite knowing the additional demands they might take on if promoted to ‘iCCM CHW’ status, ‘basic CHWs’ still expressed a longing to receive the additional iCCM responsibilities because of the idea that it will improve their quality of life overall.

### Community perceptions and rumours

Community members valued all CHWs for volunteering their time. However, when they had a sick child, they expressed an overall preference for ‘iCCM CHWs’ since they appreciated the convenient drug access and immediate curative results offered by ‘iCCM CHWs’. Hence, community members reported shifting allegiance towards ‘iCCM CHWs’ and away from ‘basic CHWs’. This shift in allegiance was also recognized by the CHWs.

Community provocations appear to have notably influenced CHW self-esteem. For example, one ‘basic CHW’ reported feeling generally satisfied in his/her role as a ‘basic CHW’ until an encounter with community members who pointed out his/her lack of additional iCCM training. This encounter reportedly made the CHW feel “small”. In parallel, an ‘iCCM only CHW’ reportedly felt content with his/her iCCM role as a drug distributor until a caregiver pointed out better efficiency of ‘iCCM plus mobile CHWs’ in other areas, since they also had lamps for treating children during the night.

Our data suggest that community member perceptions often resulted from misinformation about the iCCM intervention. For example, many informants across all participant groups, particularly Local Council Leaders and caregivers, did not display a clear understanding of why some CHWs were iCCM-trained while others were not. It is possible that this lack of understanding gave rise to the various iCCM intervention-related rumours that were reported by all informants. Oftentimes these rumours were hurtful to the reputation of certain CHW categories. For example, one reported rumour was that ‘basic CHWs’ were not iCCM-trained because they were disinterested in attending training workshops. Other reported rumours were that ‘iCCM CHWs’ were receiving secret salaries, were more competent, or were prioritized for future CHW programs.

### Social status and equity

Our data indicate that ‘iCCM CHWs’ appear to have acquired a higher social status than ‘basic CHWs’ within their communities. According to some informants, ‘iCCM CHWs’ were sometimes called ‘doctor’ or ‘nurse’, or given spotlighted introductions during special village occasions. One ‘basic CHW’ discussed his/her envy for the changed image of ‘iCCM CHWs’ in a spiritual context:

*“If a parent brings a sick child to you and you treat him and he gets well*, *the parent will thank you and say*, *“God bless you.” So the more they say it*, *the more God blesses you.”*

Since ‘iCCM CHWs’ also restocked drugs and referred very sick children to health centres, they had a higher interaction with health workers than ‘basic CHWs’, and thus reported a stronger connection to health centres and the health system in general. Some ‘iCCM CHWs’ even reported expedited service at the health centre for personal and family health issues. Conversely, ‘basic CHWs’ reported feeling disconnected from health centres and thought their ‘basic CHW’ status would lead to fewer opportunities within future CHW programs and trainings.

A strong desire for equity amongst all CHWs was a commonly recurring theme that emerged from all FGDs with CHWs. Interestingly, ‘basic CHWs’ did not discuss a particular determination for receiving either iCCM or mobile training per se; they reported just wanting anything additional “*to make things equal*”. Furthermore, it appears that the desire for additional training or tools occurred only after a realization that things were not equal or fair. For example, one ‘basic CHW’ in an ‘iCCM only’ area admitted that he/she was generally satisfied with his/her circumstances simply because the ‘iCCM CHWs’ in their area did not end up receiving mobile phones like they had hoped. In this way, they were equal in that both groups did not get what they wanted.

### Cooperation and team dynamics

Despite the emerging themes of segregation and inequity, all CHWs reported generally good personal relationships with their CHW peers, both inside and outside of their volunteer tasks. No study informant expressed any major antagonism and most expressed support for one another. Feelings of bitterness regarding iCCM circumstances were generally kept to the individual and not expressed as public confrontations. Frustrations between ‘iCCM CHWs’ and ‘basic CHWs’ varied between individuals and groups. For example, one ‘basic CHW’ criticized ‘iCCM CHWs’ for abandoning their basic health promotion tasks. On the other hand, ‘iCCM CHWs’ from the same area felt that ‘basic CHWs’ were acting indifferently. In a separate community, all CHWs reported getting along and even tried to share the workload where possible, which was attributed to the unique self-organization of non-segregated sub-teams within the community; a ‘basic CHW’ described the process as follows:

*“When we were forming small groups*, *we never categorized that let one group be comprised of members with no drugs and another to be comprised of members with drugs. Instead we all mixed up . . . We even put there some small fee [that] we contribute [to]*, *and at the end we keep giving each member [a portion of the contributions] considering the rules governing the group.”*

Interestingly, ‘iCCM CHWs’ from another community expressed compassion for the unhappiness of their ‘basic CHW’ colleagues, and suggested ways to lessen the segregation, such as training all CHWs in iCCM even if drug supplies were only assigned to a few. Other CHW informants suggested hosting a sub-county recognition event to unite all CHWs.

## Discussion

Providing only some CHWs with iCCM training, materials, and support in our iCCM pilot study unexpectedly and negatively impacted the self-esteem of ‘basic CHWs’ who carried out important health duties but did not receive the iCCM opportunity. Our data suggest that there is a positive correlation between the self-esteem of CHWs and the quantity of training or materials they received. For example, ‘iCCM plus mobile CHWs’ expressed the overall highest self-esteem because they received both iCCM and mobile phone training, whereas ‘iCCM only CHWs’ expressed feeling slightly lower self-esteem because they did not have the advantage of mobile phones. Likewise, ‘basic CHWs’ in ‘iCCM only’ areas expressed inferiority to their iCCM peers, yet ‘basic CHWs’ in ‘iCCM plus mobile’ areas expressed even lower self-esteem because they were without both iCCM and mobile phone training.

Since CHW self-esteem is linked to CHW motivation [16], future iCCM intervention policies should consider a more equal distribution of training and materials amongst the different intervention groups in order to establish a less segregated and more equitable working environment. Allocating equipment and tools to only a few CHWs can even cause those left out to refuse the project [3]. Other potentially esteem-promoting and motivational strategies include a more unified distribution of training certificates and the creation of CHW recognition events [3,10], as suggested by CHWs themselves in our study. As well, more efforts should be taken to ensure that community members understand the objective of the intervention program being implemented, since misguided community perceptions seem to have exacerbated feelings of segregation amongst CHWs.

Although our allocation of materials created a negative influence on the self-esteem of individual CHWs, it did not appear to necessarily result in poor CHW team functioning. CHW cooperation remained particularly strong amongst the team of CHWs that re-organized themselves into non-segregated sub-teams, suggesting that if CHW roles within a village team vary, team structure may be modified to maximize positive team dynamics. Literature related to CHW team functioning has demonstrated that teams are less dismembered and more pleased with their work when there is a co-responsibility for tasks and no hierarchical treatment of workers [17,18]. In any case, it is clear that at least one CHW team was able to navigate the varied roles to create a more equal and equitable work environment.

Importantly, issues of CHW motivation and long term team functioning should be considered together and require further assessment. The generally good CHW team cooperation observed in our study area may not persist over the longer term since even the slightest case of demotivation amongst CHWs can be detrimental to team functioning. If demotivation leads to deep-seeded resentment of peers, development partners, or the national program, credibility of future community health programs may also be damaged. Demotivated CHWs may lead to program unproductivity or even dropouts [10,11,19,16], hampering an otherwise potentially successful program. It is also somewhat disheartening to observe amongst ‘basic CHWs’ an insistent yearning for a drug distribution role, and to observe amongst ‘iCCM CHWs’ a strong motivation seemingly dependent on their more curative role. The curative role seemed to coincide with declining importance of the health promotion role; a pattern which could have detrimental consequences on overall CHW program success. Poor motivation towards health promotion starkly contrasts findings from previous study areas, where CHWs in health promotion-only roles were highly motivated, and strongly linked their health promotion activities with improved health within their communities [12].

According to Ugandan iCCM program guidelines, the national vision is for all CHWs to be eventually iCCM-trained [14]. As such, training just two CHWs per village is an interim measure and inequity issues raised in this study may be short-lived. However, if more than two CHWs practice iCCM, ensuring adequate patient-load to ensure maintenance of iCCM skills would still need to be addressed. Also interesting in our study was that the majority of villages had only two or three CHWs, when national guidelines called for an average of five CHWs per village. This may have exacerbated the division of those ‘with drugs’ and those ‘without’ since some ‘basic CHWs’ may have felt isolated when almost all others in their monthly meeting groups had treatment abilities. In any case, low self-esteem and segregation themes emerging from our intervention provide insight into two critical program operational issues: 1) potential risks associated with selective ‘opportunities’ (even when ‘non-financial’) within the CHW team setting, and 2) a relatively lowered value of a CHW health promotion role compared to a curative role.

Ours was a small study documenting specific issues perceived within a small, defined population. Added value might be gained by having a greater variety and number of community member perspectives. Other limitations encountered upon analysis of our data were: 1) analysis was conducted in English, which potentially limited cultural and language-sensitive responses, and 2) transcriptions did not distinguish which informant was speaking, making it unclear if opinions were equally shared throughout the focus groups or resulting from dominant speakers.

## Conclusion

Although the iCCM intervention increased the proportion of children receiving treatment for common illnesses, this pilot study raises several important operational considerations for Ugandan programmers prior to iCCM national scale-up. First, a more equal distribution of training and materials amongst CHWs should be considered. With respect to CHW training, this can be done by adjusting the optimal number of ‘iCCM CHWs’ per village in the initial program rollout, selecting fewer ‘basic CHWs’ per village, or training more ‘iCCM CHWs’. Second, guidelines ensuring clear community sensitization at the time of ‘basic CHW’ and ‘iCCM CHW’ selection must be clear and widely disseminated, including guidelines for how CHWs liaise with health facilities and staff. Third, other opportunities to promote and support good self-esteem and the health promotion role for all CHWs, is necessary. Finally, CHW retention over time should be studied including comparisons of CHWs in a variety of roles.

The lessons learned from this study have and will be shared with Ugandan policymakers; however the lessons learned are also applicable beyond Uganda. Segregation resulting from ‘iCCM’ categories of CHWs juxtaposed to ‘basic CHWs’ provides a unique opportunity (and a control group of sorts) to appreciate the strong CHW motivation that accompanies adding both materials and training opportunities to basic CHW roles. Such motivation should be tapped in positive ways. As well, our experience can inform CHW planners to carefully consider the perceived ‘material’ benefits that might be offered to all or just to a select group of CHWs. Non-financial incentives can have both positive and negative impacts on CHW program sustainability. Since CHWs are key intercessors in community intervention programs, organizations and governments must be mindful of how to encourage CHW solidarity to improve CHW motivation and retention, and thus bring CHW programs a step closer to achieving sustainability.

## List of abbreviations

CHW: community health worker; DFATD: Foreign Affairs, Trade and Development Canada; FGD: focus group discussion; GHRI: Global Health Research Initiative; HCU: Healthy Child Uganda; iCCM: integrated community case management; IDRC: International Development Research Centre; KII: key informant interview; MoH: Ministry of Health

## Competing interests

The authors declare that they have no competing interests.

## Authors' contributions

TK, JK, and JLB initiated the concept for the study. HFGM developed the tools for the study and performed data analysis, with guidance from DLB. HFGM supervised field mobilization and TK participated in data collection. HFGM, DLB, JK, and JLB participated in the interpretation and the writing of the manuscript. All authors read and approved the final manuscript.

## Declarations

The publication costs associated with this article are funded by Foreign Affairs, Trade and Development Canada and the International Development Research Centre through the Global Health Research Initiative.

This article has been published as part of *BMC Health Services Research* Volume 14 Supplement 1, 2014: Uptake and impact of research for evidence-based practice: lessons from the Africa Health Systems Initiative's research component. The full contents of the supplement are available online at http://www.biomedcentral.com/bmchealthservres/supplements/14/S1
